# Central Role of Core Binding Factor β2 in Mucosa-Associated Lymphoid Tissue Organogenesis in Mouse

**DOI:** 10.1371/journal.pone.0127460

**Published:** 2015-05-22

**Authors:** Takahiro Nagatake, Satoshi Fukuyama, Shintaro Sato, Hideaki Okura, Masashi Tachibana, Ichiro Taniuchi, Kosei Ito, Michiko Shimojou, Naomi Matsumoto, Hidehiko Suzuki, Jun Kunisawa, Hiroshi Kiyono

**Affiliations:** 1 Division of Mucosal Immunology, Department of Microbiology and Immunology, The Institute of Medical Science, The University of Tokyo, 4-6-1 Shirokanedai, Minato-ku, Tokyo, 108–8639, Japan; 2 Laboratory of Vaccine Materials, National Institutes of Biomedical Innovation, Health and Nutrition, 7-6-8 Saito-asagi, Ibaraki-city, Osaka, 567–0085, Japan; 3 Division of Virology, Department of Microbiology and Immunology, The Institute of Medical Science, The University of Tokyo, 4-6-1 Shirokanedai, Minato-ku, Tokyo, 108–8639, Japan; 4 Laboratory for Transcriptional Regulation, RIKEN Center for Integrative Medical Sciences (IMS-RCAI), 1-7-22, Suehiro-cho, Tsurumi-ku, Yokohama, 230–0045, Japan; 5 Department of Molecular Bone Biology, Graduate School of Biomedical Sciences, Nagasaki University, 1-7-1 Sakamoto, Nagasaki, 852–8588, Japan; 6 International Research and Development Center for Mucosal Vaccines, The Institute of Medical Science, The University of Tokyo, Tokyo, Japan; 7 Graduate School of Pharmaceutical Sciences, Osaka University, Osaka, Japan; 8 Department of Microbiology and Immunology, Kobe University School of Medicine, Kobe, Japan; 9 Graduate School of Medicine, The University of Tokyo, Tokyo, Japan; 10 Department of Medical Genome Science, Graduate School of Frontier Science, The University of Tokyo, Chiba, Japan; McGill University, CANADA

## Abstract

Mucosa-associated lymphoid tissue (MALT) is a group of secondary and organized lymphoid tissue that develops at different mucosal surfaces. Peyer’s patches (PPs), nasopharynx-associated lymphoid tissue (NALT), and tear duct-associated lymphoid tissue (TALT) are representative MALT in the small intestine, nasal cavity, and lacrimal sac, respectively. A recent study has shown that transcriptional regulators of core binding factor (Cbf) β2 and promotor-1-transcribed Runt-related transcription factor 1 (P1-Runx1) are required for the differentiation of CD3^−^CD4^+^CD45^+^ lymphoid tissue inducer (LTi) cells, which initiate and trigger the developmental program of PPs, but the involvement of this pathway in NALT and TALT development remains to be elucidated. Here we report that Cbfβ2 plays an essential role in NALT and TALT development by regulating LTi cell trafficking to the NALT and TALT anlagens. *Cbfβ2* was expressed in LTi cells in all three types of MALT examined. Indeed, similar to the previous finding for PPs, we found that *Cbfβ2^−/−^* mice lacked NALT and TALT lymphoid structures. However, in contrast to PPs, NALT and TALT developed normally in the absence of P1-Runx1 or other Runx family members such as Runx2 and Runx3. LTi cells for NALT and TALT differentiated normally but did not accumulate in the respective lymphoid tissue anlagens in *Cbfβ2^−/−^* mice. These findings demonstrate that Cbfβ2 is a central regulator of the MALT developmental program, but the dependency of Runx proteins on the lymphoid tissue development would differ among PPs, NALT, and TALT.

## Introduction

The developmental program of secondary lymphoid tissues is initiated by interaction between hematopoietic lymphoid tissue inducer (LTi) cells and stromal lymphoid tissue organizer (LTo) cells [1]. Differentiation of LTi cells, which surface phenotype are characterized as CD3^−^CD4^+^CD45^+^ cells occurs in the fetal liver [2–4] and is controlled by transcriptional regulators of inhibitor of DNA binding/differentiation (Id) 2 [5, 6] and retinoic acid-related orphan receptor (ROR) γt [7, 8]. Neuron-derived retinoic acid induces the initial expression of C-X-C motif chemokine ligand (CXCL) 13 in LTo cells [9], which express vascular cell adhesion molecule (VCAM)-1 and intercellular adhesion molecule (ICAM)-1 [10], thereby inducing migration of LTi cells into the lymphoid tissue anlagen. Signals mediated by CXCL13 and C-X-C motif chemokine receptor (CXCR) 5 cause latent α4β1 integrin in the LTi cellular membrane to change to the active form, thereby ensuring stable interaction between LTi cells and LTo cells[11]. At the anlagen site, LTi cells are activated via interleukin-7 receptor (IL-7R)–α or receptor activator of NF-κB (RANK)–mediated signals, which induce expression of membrane-bound lymphotoxin (LT) α1β2 heterotrimer on the LTi cell surface [12]. LTi cell–derived LTα1β2 stimulates LTβR-expressing LTo cells through alternative NF-κB pathways, which induces production of large amounts of lymphoid chemokines (e.g., CXCL13, CCL19, and CCL21) and adhesion molecules (e.g., VCAM-1 and ICAM-1)[13]. This process induces migration of large numbers of LTi cells as well as conventional leukocytes into lymphoid tissue anlagen. The cytokine and chemokine signals, triggered by LTi cells and LTo cells, form a positive feedback loop that promotes the generation of the organizing center of lymphoid tissues [1].

The mucosal surface is equipped with mucosa-associated lymphoid tissue (MALT), which is capable of sampling luminal antigens, through the action of M cells located in the follicle-associated epithelium, to initiate antigen-specific immune responses [14]. MALT includes PPs in the small intestine, nasopharynx-associated lymphoid tissue (NALT) in the nasal cavity, and tear duct-associated lymphoid tissue (TALT) in the lacrimal sac [15]. Development pathways of PPs has been extensively characterized and has been shown to share a common cellular and molecular organogenesis program with the peripheral lymph nodes in which CXCL13-CXCR5 and LTα1β2-LTβR-NIK axes play a central role [1]. In contrast, organogenesis of NALT and TALT does not utilize the above pathway. Initiation of NALT and TALT genesis occurs after birth, whereas that of PPs takes place during late embryogenesis [14, 16]. Although LTi cells are the first hematopoietic population seen in all MALT anlagens, the CXCL13-mediated signal is not essential for NALT or TALT development [16, 17]. Initiation of NALT and TALT genesis occurs independently of IL-7R–LTα1β2-LTβR-NIK signaling [16, 18]. Moreover, requirement for transcriptional regulators is different among PPs, NALT, and TALT. While development of PPs depends on both Id2 and RORγt, NALT formation is absent only in *Id2*-deficient mice and is independent of RORγt [18, 19]. Intriguingly, development of TALT does not require either Id2 or RORγt [16]. Indeed, expression of mRNA encoding Id2 and RORγt was not detected in LTi cells isolated from TALT anlagen, whereas the LTi cells isolated from small intestine express both mRNAs [16]. These results indicate that LTi cells are generally involved in MALT development but biological nature of LTi cells for distinct types of MALT, (PPs, NALT and TALT) could be different.

The transcriptional complex of Runt-related transcription factor protein and core binding factor (Cbf) has recently been shown to be involved in PP development [20]. Cbfβ molecules do not bind to DNA, but they enhance the binding activity of Runx proteins to DNA in an allosteric manner [21, 22]. The *Cbfβ* gene comprises 6 exons and is transcribed into three different mRNA transcripts by alternative splicing (*Cbfβ1–3*) [23, 24]. *Cbfβ2* mRNA is the full-length type, whereas *Cbfβ1* mRNA shows a 31-bp deletion in exon V [25]. *Cbfβ3* mRNA lacks exon V, which results in deficiency of binding activity of Cbfβ3 protein with Runx proteins [25, 26]. *Cbfβ* ^−*/*−^ mice show impairment of fetal liver hematopoiesis and die between embryonic days (E) 11.5 and E14.5 [27, 28]. A previous study of mice with an isoform-specific deletion of the *Cbfβ* gene (i.e., *Cbfβ1* ^−*/*−^ or *Cbfβ2* ^−*/*−^) revealed that PP organogenesis is disrupted in *Cbfβ2* ^−*/*−^ mice but not *Cbfβ1* ^−*/*−^ mice [20]. Runx proteins are key transcription factors in major tissue developmental pathways, and are encoded by three distinct genes. Each of the three genes, *Runx1*, *Runx2 and Runx3* is transcribed by two types of promoters (P1, distal; P2, proximal) [29]. *Runx1* ^−/−^ mice show a lack of definitive hematopoiesis, similar to that observed in *Cbfβ* ^−*/*−^ mice, resulting in embryonic lethality between E12.5 and E14.5 [30, 31]. Mice with selective loss of promotor-1-transcribed Runx1 (P1-Runx1), hereafter reffered as to *P1-Runx1* ^−/−^ mice survive after birth but show impaired hematopoiesis. In *P1-Runx1* ^−/−^ mice, differentiation of basophils and natural killer T (NKT) cells as well as LTi cells are severely impaired, while T and B cell development is unaffected [20, 32]. When the P2 promoter activity is attenuated in the *Runx1* gene, mice die shortly after birth and development of hematopoietic progenitor cells in fetal liver is reduced [33, 34]. Expression of the *Runx2* gene is essential for osteogenesis, thereby, *Runx2* gene deficiency leads to the absence of ossification of the ribs, resulting in neonatal death soon after birth without breathing [35, 36]. Ribs lacking in ossification would not be strong enough to provide the negative pressure required for lung expansion. Without P1-Runx2 isoform, around 20% of mice, hereafter referred as to *P1-Runx2* ^−/−^ mice, survive for at least 1 week, and show diminished osteoblastic function, leading to low-turnover osteopenia in adult mice [37, 38]. *Runx3* ^−/−^ mice show a impaired differentiation of CD8^+^ cytotoxic T cells [39, 40], neurons [41, 42], and a causal relationship with gastrointestinal cancer [43–45]. Thus, heterodimeric Runx/Cbfβ transcription factor complexes play multiples important roles in the control of development of many types of cells. However, their roles in the development of NALT and TALT remain to be elucidated. In this study, we demonstrated that Cbfβ2 plays a major role in the development of NALT and TALT, and showed that the roles of Runx family proteins differ among PPs, NALT and TALT.

## Results

### Expression of Cbfβ protein in CD3^−^CD4^+^CD45^+^ LTi cells in the MALT anlagens

**Fig 1:**
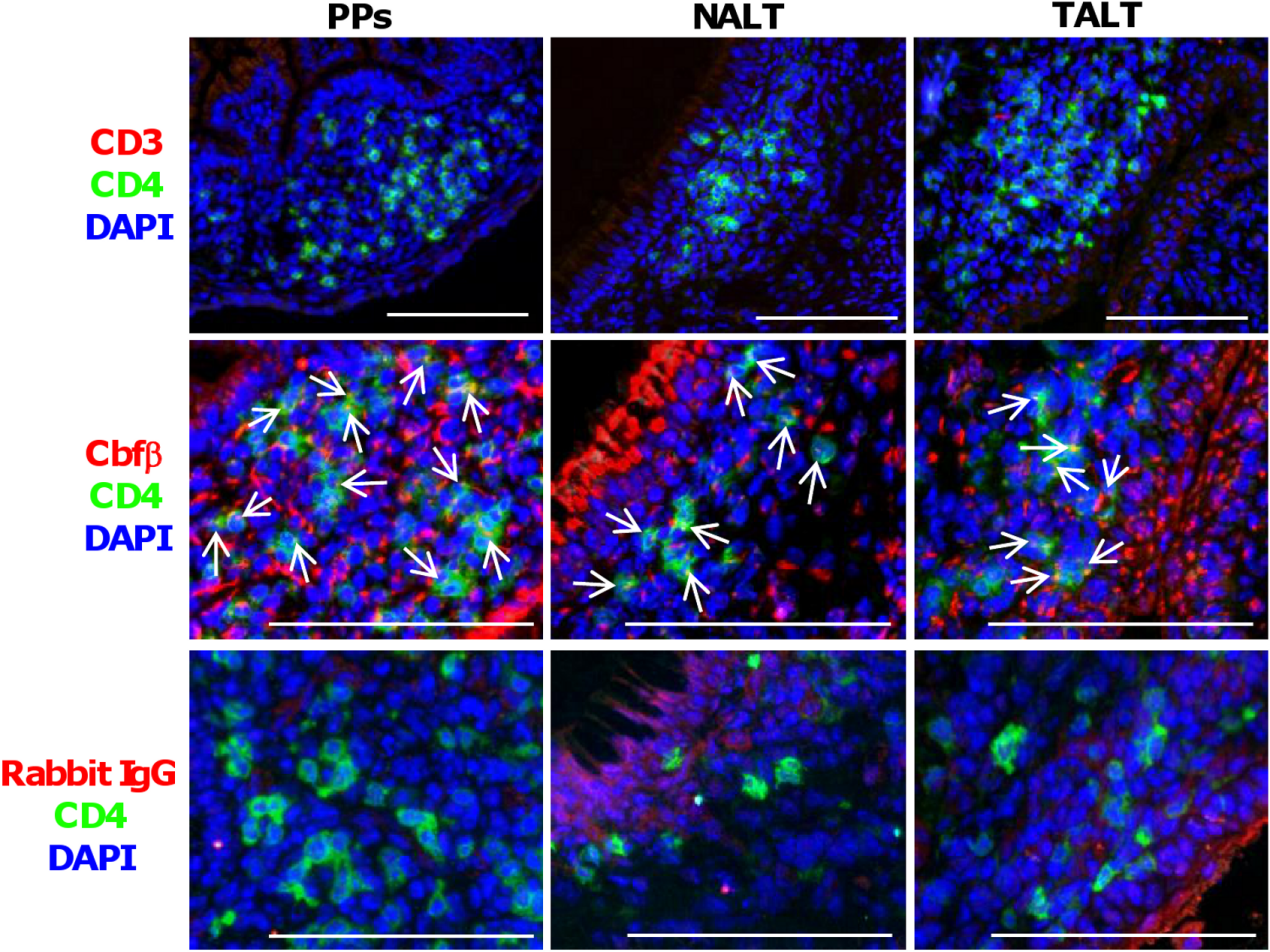
Expression of Cbfβ in MALT LTi cells. Cryostat sections of the PP, NALT, and TALT anlagens of C57BL/6 *wild-type* mice were prepared from E17 small intestine, D10 nasal tissue, and D10 tear duct, respectively. Samples were analyzed by means of fluorescence microscopy with the indicated antibodies and reagent. Arrows point to Cbfβ expression in LTi cells. Data are representative of at least three independent experiments (n = 2 mice/group). Bars, 100 μm.

Consistent with our previous reports [16–18], we detected an accumulation of CD3^−^CD4^+^ LTi cells in anlagen of MALT at the period of MALT organogenesis in each case, PP anlagen of the small intestine on E17 and NALT and TALT anlagens on neonatal day 10 (D10) (Fig 1). We next conducted an immunohistochemical analysis of Cbfβ expression by staining the tissues with rabbit anti-mouse Cbfβ monoclonal antibody (mAb). Positive signals of Cbfβ were detected in the lymphoid tissue anlagens of NALT and TALT as well as PPs (Fig 1). CD3^−^CD4^+^ LTi cells were found to express Cbfβ (Fig 1). Tissues stained with rabbit IgG control Ab detected no positive signals in LTi cells, ensuring the specificity of the Cbfβ signals (Fig 1).

### Gene expression of Cbfβ and Runx isoforms in CD3^−^CD4^+^CD45^+^ LTi cells in the MALT anlagens

**Fig 2:**
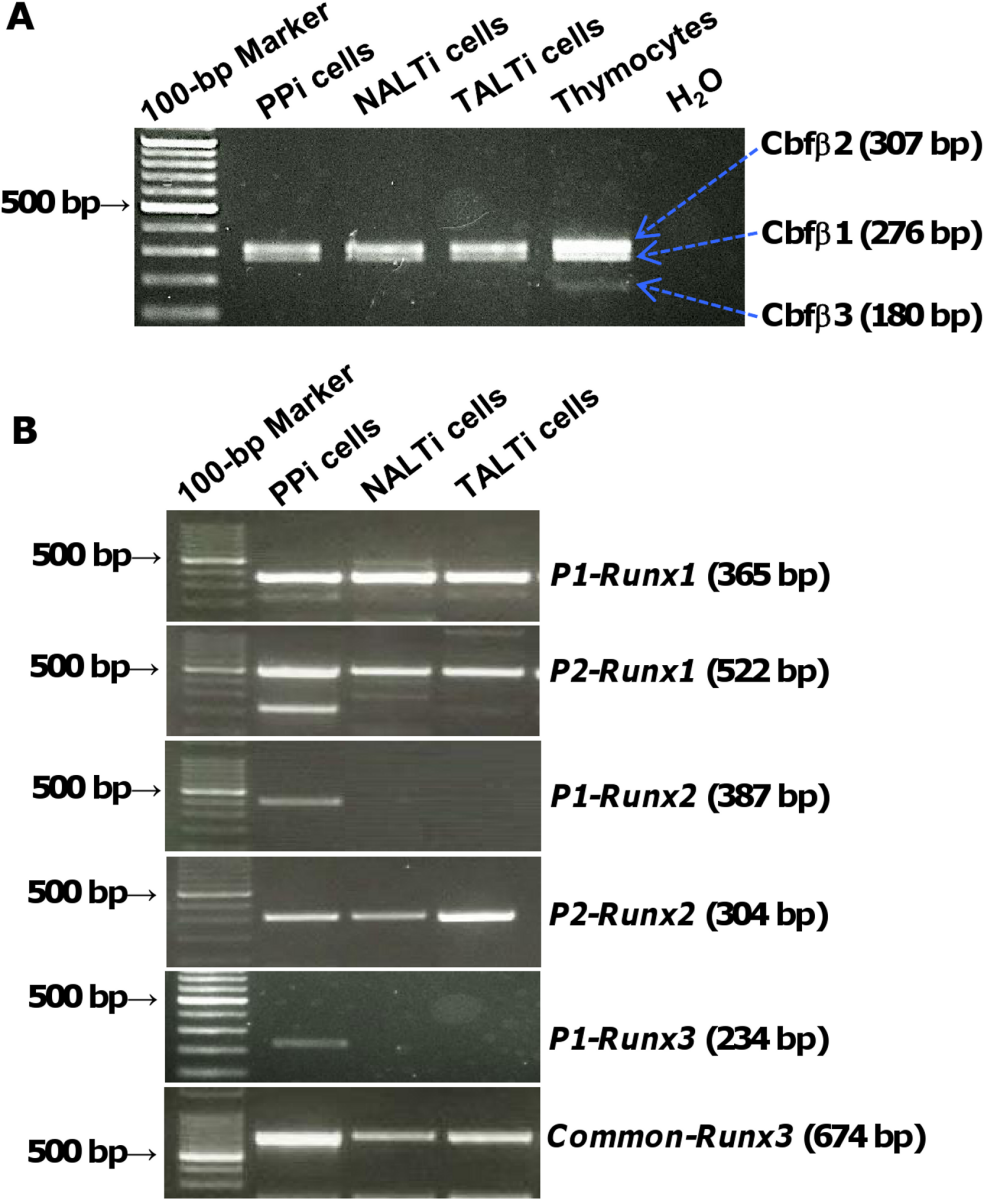
Expression of isoform-specific *Cbfβ* and *Runx* mRNA by MALT LTi cells. CD3^−^CD4^+^CD45^+^ LTi cells were isolated from E17 small intestine (PPi cells), D10 nasal tissue (NALTi cells), and D10 tear duct (TALTi cells). Expression of the *Cbfβ* (A) and *Runx* isoforms (B) was examined by means of RT-PCR. Data are representative of 2 independent experiments (n = 18–20 mice/group). Thymocytes were used as a positive control for *Cbfβ1*,*2* and *3* isoforms (arrows in A). H_2_O was used as a negative control of PCR.

To determine whether LTi cells in each MALT anlagen expressed the *Cbfβ2* gene, CD3^−^CD4^+^CD45^+^ cells were isolated from E17 small intestine, D10 nasal cavity, and D10 tear duct as PP inducer cells (PPi cells), NALT inducer cells (NALTi cells), and TALT inducer cells (TALTi cells), respectively. The primers used in PCR amplification detect all three *Cbfβ* mRNA isoforms but could distinguish each mRNA by the length of the PCR products (*Cbfβ1*, 276 bp; *Cbfβ2*, 307 bp; *Cbfβ3*, 180 bp). This RT-PCR analysis revealed that *Cbfβ1* and *Cbfβ2* but not *Cbfβ3* transcripts were expressed in PPi, NALTi, and TALTi cells (Fig 2A). We next sought to determine whether LTi cells express *Runx* family genes. When we used isoform-specific primers, we found that *Runx1* was expressed in PPi, NALTi, and TALTi cells, regardless of *P1* or *P2* promoter usage (Fig 2B). In addition, we found that *P2-Runx2* mRNA was present in all three inducer cell types (Fig 2B). In contrast, the expression of *P1-Runx2* and *P1-Runx3* was absent in NALTi and TALTi cells, but present in PPi cells (Fig 2B). When we used primers that commonly amplify both *P1-* and *P2-Runx3*, expression of *Runx3* mRNA was detected in NALTi and TALTi cells, suggesting that NALTi and TALTi cells are likely to express *P2-Runx3* mRNA (Fig 2B).

### Impairment of NALT and TALT organogenesis in *Cbfβ2* ^−/−^ mice

**Fig 3:**
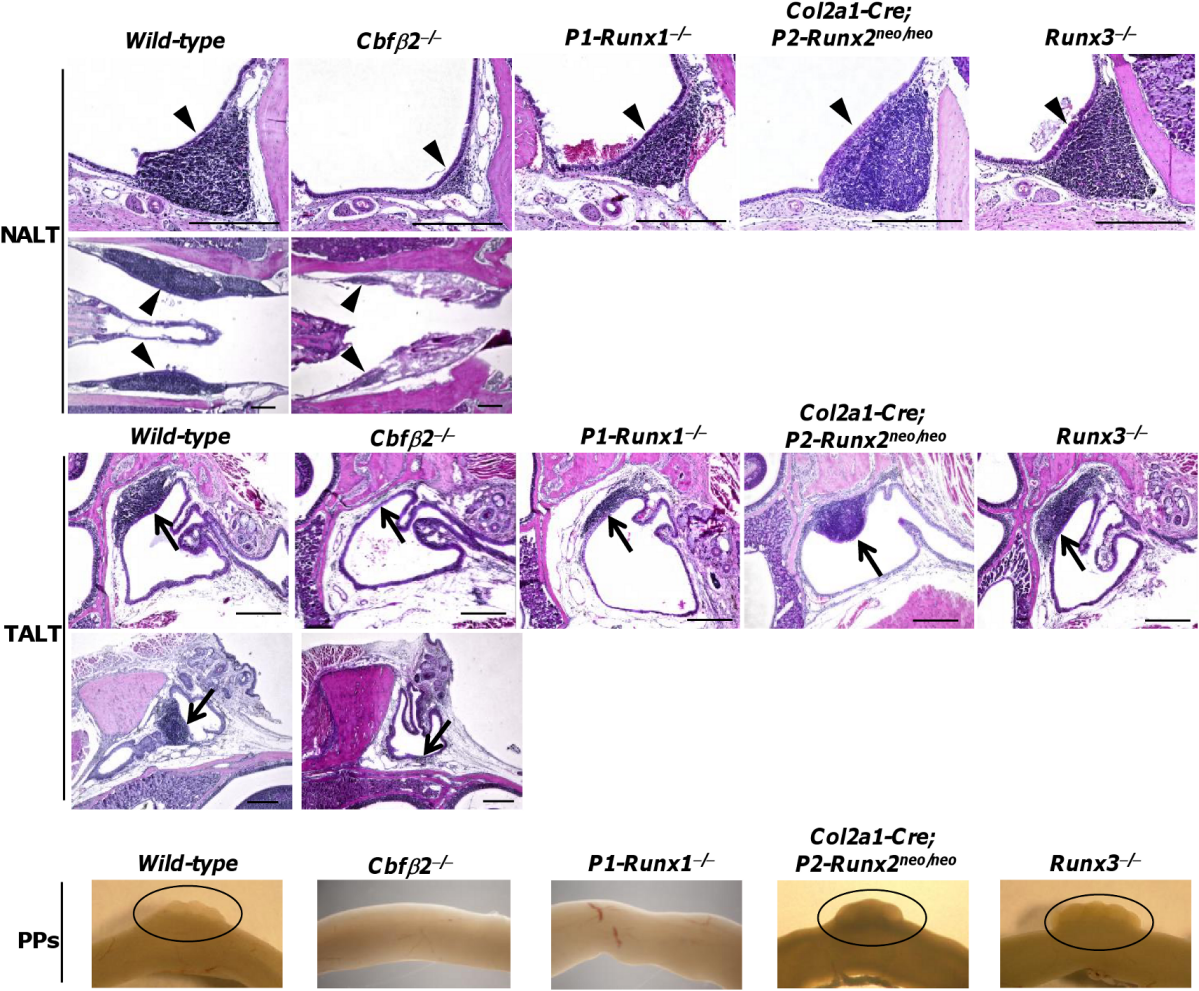
Impairment of NALT and TALT organogenesis in *Cbfβ2* ^−/−^ mice. NALT and TALT development was examined by H&E staining of paraffin-embedded tissue sections of 8-week- to 16-week-old adult mice. PP structure of 8-week- to 1-year-old adult mice was observed under microscopy. Genesis of NALT and TALT of *wild-type* mice and *Cbfβ2* ^−/−^ mice were evaluated with coronal (upper panels) and horizontal (lower panels) sections. NALT and TALT of *P1-Runx1* ^−/−^ mice, *Col2a1-Cre; P2-Runx2* ^*neo/neo*^ mice and *Runx3* ^−/−^ mice were examined with coronal sections. Arrowheads and arrows point to NALT and TALT, respectively. Circles indicate the presence of PP structure. Data are representative of at least five independent experiments (n = 3 mice/group). Bars, 300 μm.

Expression of the *Cbfβ* and *Runx* gene in NALTi and TALTi cells raised the possibility that Cbfβ/Runx complex might involve in NALT and TALT organogenesis. Strikingly, histological analysis of coronal and horizontal sections of head tissue revealed that lack of Cbfβ2 isoform resulted in a severe defects for NALT and TALT organogenesis (Fig 3). Thus, hematoxylin & eosin (H&E) staining of the sections indicated that accumulation of mononuclear cells was diminished in both NALT and TALT regions of *Cbfβ2* ^−*/*−^ mice (Fig 3). *Cbfβ2* ^−*/*−^ mice also lacked PPs structure as reported previously[20], suggesting that Cbfβ2 plays a major role in MALT organogenesis. In contrast, *Cbfβ1* ^−*/*−^ mice showed normal development of NALT and TALT (S1 Fig), as is the case with PPs [20]. Given a previous report showing that generation of PPs depends P1-Runx1 [20], we next examined NALT and TALT development in *P1-Runx1* ^−*/*−^ mice, and found that development of both NALT and TALT were present in *P1-Runx1* ^−*/*−^ mice while PPs structure was absent (Fig 3). Thus, P1-Runx1 isoform is not essential for generation of NALT and TALT, suggesting that other Runx protein(s) might be involved in NALT and TALT organogenesis. Because NALTi and TALTi cells expressed *P2-Runx2* and *P2-Runx3* in addition to *P1-Runx1*, we next examined mice with altered *Runx2* and *Runx3* expression. In *Runx3*-deficient mice that lack both *P1- and P2-Runx3* gene expression [43], we could find both NALT and TALT structures (Fig 3). We recently generated *P2-Runx2* ^*neo/neo*^ mice in which normal *P2-Runx2* gene expression was impeded by causing *neo* gene cassette insertion into an intron between exon 2 and 3, hereafter referred as to *Col2a1-Cre; P2-Runx2* ^*neo/neo*^ mice[46]. In *Col2a1-Cre; P2-Runx2* ^*neo/neo*^ mice, Runx2 expression is rescued only in Col2a1-expressing bone-related cells, therefore, *P2-Runx2* gene expression was depressed in non-bone-related cells including LTi cells. In these mice, development of NALT and TALT was also observed (Fig 3). Both *Col2a1-Cre; P2-Runx2* ^*neo/neo*^ mice and *Runx3*-deficient mice possessed PP structure (Fig 3). These results indicate that specific isoforms of Runx proteins were not required for NALT and TALT development.

### Cbfβ2 regulates NALT and TALT development, but not via RORγt induction

**Fig 4:**
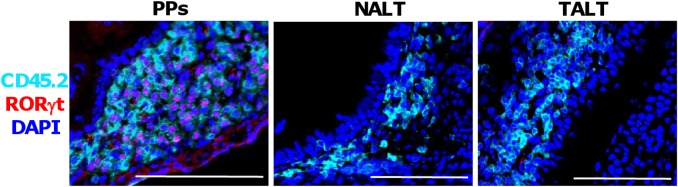
Immunohistochemical analysis of the expression of RORγt in MALT anlagens. Cryostat sections of PP, NALT, and TALT anlagens of C57BL/6 *wild-type* mice were prepared from E17 small intestine, D10 nasal tissue, and D10 tear duct, respectively. Samples were analyzed by means of fluorescence microscopy with the indicated antibodies and reagent. Data are representative of at least three independent experiments (n = 2 mice/group). Bars, 100 μm.

In PP genesis, Cbfβ2 plays an important role in inducing the expression of the *Rorγt* gene, which encodes a transcriptional factor essential for the differentiation of PPi cells [20]. Consistent with this finding, our immunohistochemical analysis of *wild-type* mice revealed that RORγt was present in PP anlagen (Fig 4). In contrast, there were no positive signals for RORγt in NALT or TALT anlagen, suggesting that differentiation of NALTi and TALTi cells occurs independently from RORγt production even though Cbfβ2 was expressed in both these inducer cell types (Fig 4). These results are consistent with previous reports that RORγt is dispensable for NALT and TALT development [16, 19]. To address whether NALTi and TALTi cells were present in *Cbfβ2* ^−*/*−^ mice, we performed FACS analysis. The results revealed that CD3^−^CD4^+^CD45^+^ NALTi and TALTi cells were present in the nasal tissues and tear duct compartment of *Cbfβ2* ^−*/*−^ mice (S2 Fig), indicating that Cbfβ2 might play critical roles in NALT and TALT development independently from the known LTi cell differentiation (e.g., PPi cells).

### Defects in LTi cell trafficking to NALT and TALT anlagens in *Cbfβ2* ^−/−^ mice

**Fig 5:**
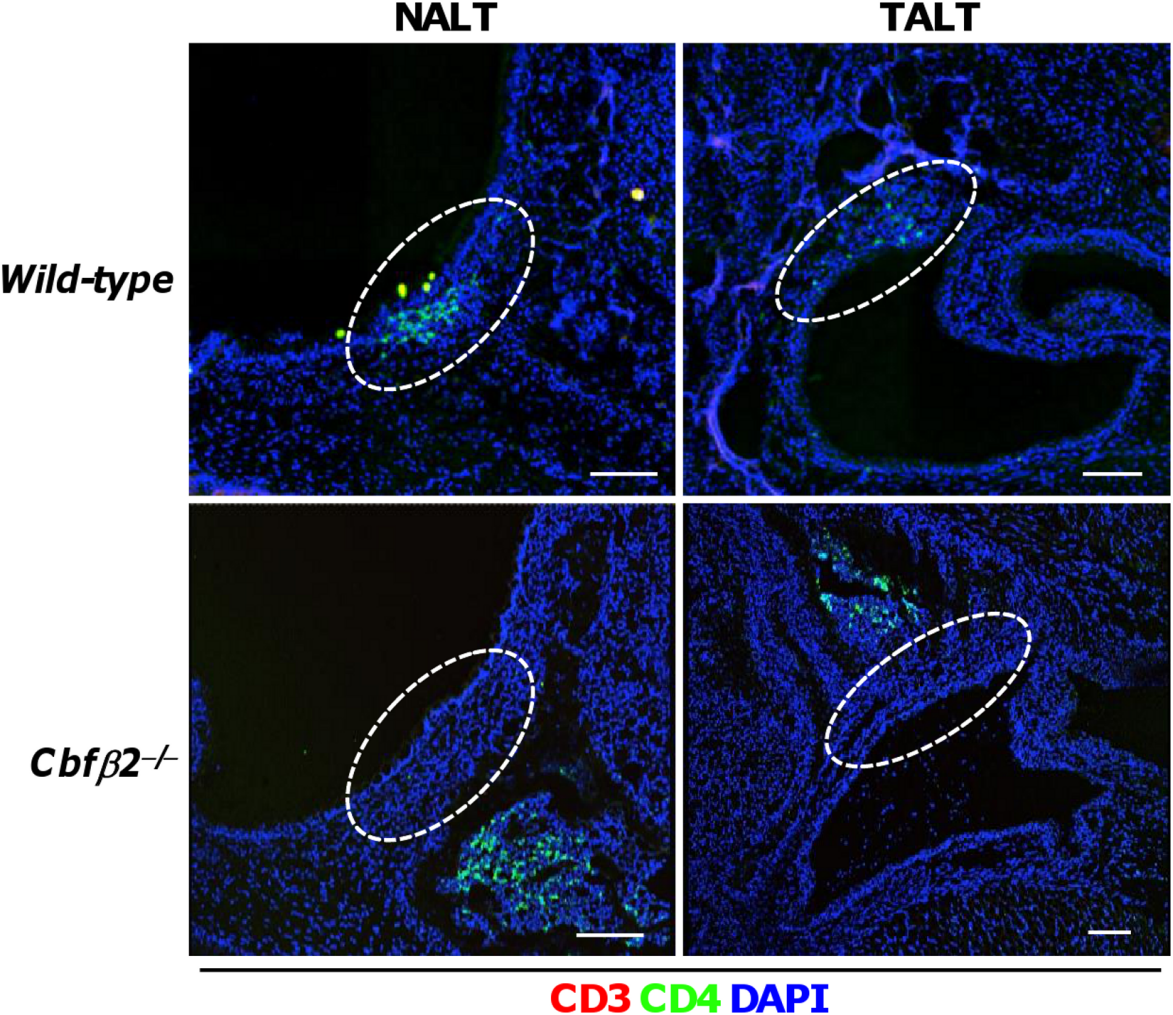
Abnormal localization of NALTi and TALTi cells in *Cbfβ2* ^−/−^ mice. NALT and TALT anlagens were analyzed by means of fluorescence microscopy with the indicated antibodies and reagent. In each case, CD3^−^CD4^+^ cells were found to localize away from the anlagen in *Cbfβ2* ^−/−^ mice. Dotted lines indicate the area of each lymphoid tissue anlagen. Data are representative of at least two independent experiments (n = 3 mice/group). Bars, 100 μm.

Because accumulation of LTi cells in anlagens is an important initial process in lymphoid tissue organogenesis [1], we next asked whether NALTi and TALTi cells could migrate to and accumulate in the NALT and TALT anlagens without Cbfβ2 expression. Immunohistochemical analysis of *Cbfβ2* ^−*/*−^ mice revealed that NALTi and TALTi cells were localized away from their respective anlagens (Fig 5). The location of NALTi and TALTi cells of *Cbfβ2* ^−*/*−^ mice was likely to be nasal mucosa and tear duct lamina propria regions where CD31^+^ vessels and/or LYVE-1^+^ lymphatic vessel existed (S3 Fig). We hypothesized that NALTi and TALTi cells retained in the nasal mucosa and tear duct lamina propria regions due to defects in the expression of adhesion molecules in *Cbfβ2* ^−*/*−^ mice. Many target genes of Runx proteins have been identified in the hematopoietic cells, for instance, IL-3, GM-CSF, and CD11a [47, 48]. It is found that PPi, NALTi, and TALTi cells of *wild-type* mice expressed CD11a (S4 Fig). CD11a and CD18 form a complex called lymphocyte function associated antigen-1 (αLβ2 integrin, or LFA-1), which promotes cell-cell adhesion and activation [49]. The expression of CD18 was found on PPi, NALTi, and TALTi cells, suggesting that these LTi cells could make the LFA-1 complex (S4 Fig). However, the expression level of CD11a was not changed in *Cbfβ2* ^−*/*−^ mice (S5 Fig). The finding indicates that Cbfβ2 regulates migration of NALTi and TALTi cells independently from gene induction of CD11a. Molecular mechanism of Cbfβ2-mediated NALTi- and TALTi-cell-trafficking to respective MALT anlagen needs to be further investigated in later study.

## Discussion

Here we provide genetic evidence showing that Cbfβ2 is required for NALT and TALT development. However, biological and functional property of Cbfβ2 in the control of these two MALT tissues seemed different to those for a classical lymphoid tissue organogenesis (e.g., PP genesis). Formation of PPs requires expression of both Cbfβ2 and P1-Runx1, indicating that Cbfβ2 function as heterodimeric complexes in this process [20]. However, we found that NALT and TALT development did not require P1-Runx1, while Cbfβ2 is still necessary. Moreover, although *P2-Runx2* and *Runx3* transcripts were expressed in NALTi and TALTi cells, their encoding proteins were dispensable for development of NALT and TALT. Because we failed to detect expression of *P1-Runx2* mRNA in NALTi and TALTi cells, the remaining candidate to form a heterodimer with Cbfβ2 is only a P2-Runx1. However, mice with an attenuated P2 promoter activity in the *Runx1* gene die soon after birth [33, 34], precluding analysis of NALT and TALT development, which occurs around a week after birth. Given expression of several Runx isoforms in NALTi and TALTi cells, our results do not exclude a possibility that the lack of a specific Runx isoform in genetically altered mice can be compensated for by one or more other isoforms, while such redundancy from related Runx proteins does not work efficiently in genesis of PPs.

PP genesis is dependent on Rorγt, and the loss of Cbfβ2, but not P1-Runx1, impairs up-regulation of *Rorγt* gene expression in the PP anlagen [20]. In contrast, NALT and TALT development is independent of RORγt [16, 19]. Therefore, Cbfβ2 might play an important role in MALT development not only for induction of *Rorγt* but also in the control of an unknown factor that is essential for NALT and TALT development. Thus, we considered it was likely that Cbfβ2 contributed to NALT and TALT development by regulating LTi cell adhesion or migration to the site of lymphoid tissue anlagens, or both, through induction of the LFA-1complex. However, the expression level of CD11a (a ligand of ICAM-1) was not changed in the absence of Cbfβ2. The anlagen-specific migration of PPi cells is well characterized compared with that of NALTi and TALTi cells. PPi cells use CXCR5 for their specific migration; therefore, PPi cells in mice deficient for CXCR5 ligand (i.e., *Cxcl13* ^−/−^ mice) are present in a scattered manner in the small intestinal lamina propria region, thus failed to accumulate in the anlagen site, which eventually led to a loss of PP development [17]. In contrast, NALTi and TALTi cells do not express CXCR5, consistent with a fact that development of NALT and TALT is independent of the CXCL13-mediated LTi cell migration system [16, 17]. Essential signals for the migration of NALTi and TALTi cells have not yet been identified. Our results suggest that Cbfβ2 may contribute to a regulatory process that controls migration of NALTi and TALTi cells into their respective anlagen sites, and provide a new insights into the mechanisms of MALT LTi cell trafficking.

Collectively, our results clearly showed that Cbfβ2 plays a central and common role in MALT development by using a different partner Runx protein in genesis of distinct types of MALT such as PPs, NALT, and TALT.

## Materials and Methods

### Mice

C57BL/6 mice were purchased from Japan SLC. *Cbfβ2* ^−*/*−^, *Cbfβ1* ^−*/*−^, *P1-Runx1* ^−*/*−^, *Col2a1-Cre*; *P2-Runx2* ^*neo/neo*^, and *Runx3* ^−*/*−^ mice were generated as previously described [20, 34, 43, 46]. The protocol was approved by the Animal Care and Use Committee of the University of Tokyo (Permit Number: 19–2 and 20–28) and by the Animal Care and Use Committee of the National Institute of Biomedical Innovation (Permit Number: DS25-2R6 and DS25-3R5) in accordance with their guidelines. Daily feeding/watering were done *ad libitum*. Mice were euthanized by cervical dislocation under anesthesia with isoflurane and used for experiments. E17 embryo was used for the analysis of PP anlagen. D10 neonatal mice were used for the analysis of NALT and TALT anlagen. 8-week- to 1-year-old mice were used as adult mice for NALT, TALT and PP development. Thymocytes were prepared from 8-week-old *wild-type* mice.

### Antibodies and reagents

The antibodies and reagents used for immunohistological analysis were as follows: purified-anti-CD3 mAb (145-2C11; Biolegend), purified-anti-CD4 mAb (GK1.5; Biolegend), Alexa Fluor 647-anti-CD45.2 mAb (104; Biolegend), PE-anti-RORγt mAb (AFKJS-9; eBioscience), purified-anti-Cbfβ/PEBP2B mAb (EPR6322; abcam), rabbit IgG control Ab (Thermo Scientific), purified anti-CD31 mAb (MEC13.3; Biolegend) and biotin-anti-LYVE-1 mAb (ALY7; eBioscience). Purified antibodies were visualized by using secondary antibodies of Alexa Fluor 488- or Alexa Fluor 647-anti-armenian hamster IgG (Invitrogen), Cy3-anti-rat IgG (Jackson ImmunoResearch), and Cy3-anti-rabbit IgG (Jackson ImmunoResearch). Biotin-conjugated Ab was visualized with Alexa Fluor 488-streptavidin (Life Technologies). The antibodies and reagents used for FACS analysis were as follows: FITC-or PE-anti-CD3 mAb (145-2C11; BD Biosciences), PE-anti-CD11a mAb (2D7; BD Biosciences), APC-anti-CD4 mAb (RM4-5; BD Biosciences), FITC-anti-CD18 mAb (C71/16; BD Biosciences), BV421-anti-CD45 mAb (30-F11; Biolegend), purified-anti-CD16/32 mAb (93; Biolegend), PE-rat IgG2a control mAb (RTK2758; Biolegend) and Via-probe (BD Biosciences).

### Immunohistochemistry

Immunohistological analysis was performed as described previously [50] with some modifications. Tissues samples were frozen with liquid nitrogen in OCT compound (Tissue Tek). Freshly prepared cryostat sections (6-μm thick) were fixed with 95% ethanol (Nacalai Tesque) for 30 min at 4°C, followed by 100% acetone (Nacalai Tesque) for 1 min at room temperature. After being blocked with 2% (v/v) newborn calf serum in PBS (Nacalai Tesque) for 30 min at room temperature, samples were incubated with primary antibodies for 16 h at 4°C. Samples were then washed with 0.1% (v/v) Tween-20 (Nacalai Tesque) in PBS and then PBS, each for 5 min, followed by staining with secondary antibodies for 30 min at room temperature. Samples were washed with PBS two times, each for 5 min, followed by staining with DAPI (AAT Bioquest) for 10 min at room temperature to visualize nuclei. Finally, samples were washed twice with PBS, mounted in Fluoromount (Diagnostic BioSystems), and examined under a fluorescence microscope (BZ-9000; Keyence).

### Histology

Histological analysis was performed as described previously [18]. Briefly, tissues were fixed in 4% paraformaldehyde (Nacalai Tesque), followed by grease removal with 50% (v/v) chloroform (Nacalai Tesque) in ethanol for 2 h, and decalcification in 0.24 M solution of 2NA(EDTA/2Na) and 4NA(EDTA/4Na) (DOJINDO) for 10 days at 4°C. Paraffin sections (5-μm thick) were stained with H&E and analyzed under a microscope (BZ-9000; Keyence).

### Cell preparation and FACS analysis

CD3^−^CD4^+^CD45^+^ LTi cells were isolated as described previously [17]. In brief, cell samples were prepared from E17 intestine, D10 nasal tissue and tear duct for the analysis of PPi, NALTi and TALTi cells, respectively [16]. Isolated tissue samples were dissected with scissors and incubated in 0.5mg/ml collagenase (Wako) solution for 30 min at 37°C with stirring. Cell samples were incubated with anti-CD16/32 mAb prior to staining with respective antibodies to avoid non-specific binding. Dead cells were gate out by staining with Via-probe. FACS analysis was performed by BD FACSCanto (BD Bioscience) or BD FACSCalibur (BD Bioscience), and data were analyzed with Flowjo software (Digital Biology). LTi cells were isolated by BD FACSAria (BD Bioscience).

### RT-PCR

Total RNA was extracted and RT-PCR was conducted as previously described [51]. The sequences of primers used were as follows: *Cbfβ*, (sense) 5′-GCAAGGTATACTTGAAGGCT-3′ and (anti-sense) 5′-TGAGATCATCACCGCCACCT-3′; *P1-Runx1*, (sense) 5′-GAAACGATGGCTTCAGACAGC-3′ and (anti-sense) 5′-ATGACGGTGACCAGAGTGCC-3′; *P2-Runx1*, (sense) 5′-CTTGGGTGTGAGGCCGATCC-3′ and (anti-sense) 5′-ATGACGGTGACCAGAGTGCC-3′; *P1-Runx2*, (sense) 5′-GAGGGCACAAGTTCTATCTG-3′ and (anti-sense) 5′-GGTGGTCCGCGATGATCT-3′; *P2-Runx2*, (sense) 5′-ATGCGTATTCCTGTAGATCCGAGC-3′ and (anti-sense) 5′-GGTGGTCCGCGATGATCT-3’; *P1-Runx3*, (sense) 5′-GTCAGCGTGCGACATGGCTTCCAACAG-3′ and (anti-sense) 5′-AGCACGTCCACCATCGAGCGCACTTCGG-3′; Common *Runx3*, (sense) 5′-GGCAAGATGGGCGAGAACAG-3′ and (anti-sense) 5′-CGTAGGGAAGGAGCGGTCAA-3′.

## Supporting Information

S1 Fig Lack of dependence of NALT and TALT organogenesis on Cbfβ1 proteins. Paraffin-embedded tissue sections of 8- to 12-week-old *Cbfβ1* ^−*/*−^ mice were analyzed by means of H&E staining. NALT and TALT genesis were evaluated with coronal sections. Arrowheads and arrows point to NALT and TALT, respectively. Data are representative of at least two independent experiments (n = 3 mice/group). Bars, 300 μm.
(TIF)

S2 Fig Differentiation of NALTi and TALTi cells in *Cbfβ2* ^−*/*−^ mice. Nasal tissue and tear duct of D10 C57BL/6 *wild-type* and *Cbfβ2* ^−*/*−^ mice were analyzed by means of FACS. CD3^−^CD4^+^CD45^+^ cells were found even in the absence of Cbfβ2. Data shown are Via-prove negative live cell population. Data are representative of at least 3 independent experiments (n = 3 mice/group).
(TIF)

S3 Fig Expression of CD31 and LYVE-1 in nasal tissues and tear duct. D10 nasal tissue and tear duct of C57BL/6 *wild-type* mice were examined by means of fluorescence microscopy. CD31 and LYVE-1 positive signals were detected in the area of nasal mucosa and tear duct lamina propria. Dotted lines indicate the site of NALT and TALT anlagens. Data are representative of at least 2 independent experiments (n = 3 mice).
(TIF)

S4 Fig Expression of CD11a and CD18 in MALT LTi cells. FACS analysis of E17 intestine, D10 nasal tissue, and D10 tear duct was performed to examine the expression of CD11a and CD18 by PPi, NALTi, and TALTi cells, respectively. CD3^−^CD4^+^ cells in all three tissue types were found to express CD11a and CD18. Data shown are Via-prove negative live cell population. Data are representative of at least 2 independent experiments (n = 6 mice/group).
(TIF)

S5 Fig Normal expression level of CD11a in NALTi and TALTi cells of *Cbfβ2* ^−*/*−^ mice. FACS analysis of D10 nasal tissue and D10 tear duct of *wild-type* mice and *Cbfβ2* ^−*/*−^ mice was performed to examine the expression level of CD11a. Histgram data shown are Via-probe^−^CD3^−^CD4^+^CD45^+^ gated population. The expression level of CD11a in NALTi and TALTi cells of *Cbfβ2* ^−*/*−^ mice were not changed as compared with that of *wild-type* mice. Data are representative of at least two independent experiments (n = 6 mice/group).
(TIF)
