# Supplementary figures and images for: Central Role of Core Binding Factor β2 in Mucosa-Associated Lymphoid Tissue Organogenesis in Mouse
Source: PLoS One. 2015 May 22;10(5):e0127460. doi: 10.1371/journal.pone.0127460 (PMC4441428; doi:10.1371/journal.pone.0127460)

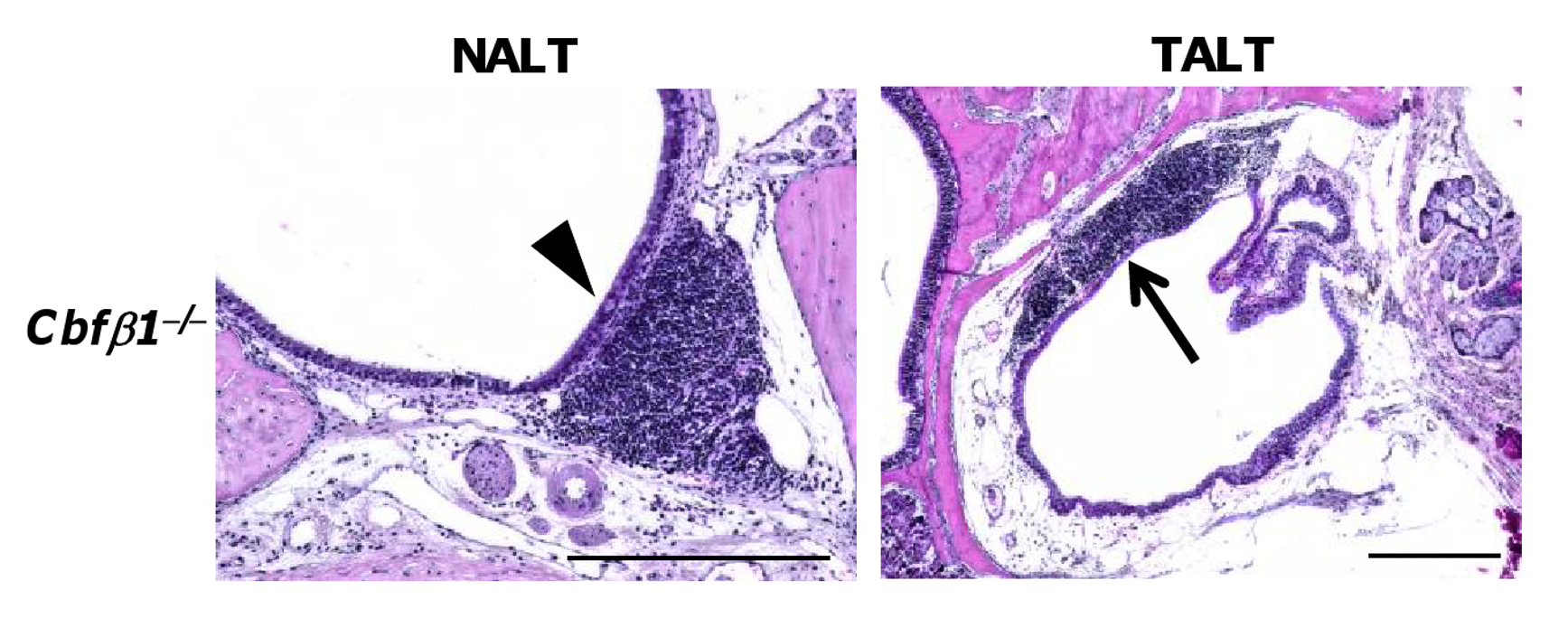

Supplement: S1 Fig — Paraffin-embedded tissue sections of 8- to 12-week-old Cbfβ1 −/− mice were analyzed by means of H&E staining. NALT and TALT genesis were evaluated with coronal sections. Arrowheads and arrows point to NALT and TALT, respectively. Data are representative of at least two independent experiments (n = 3 mice/group). Bars, 300 μm. (TIF) [file pone.0127460.s001.tif]

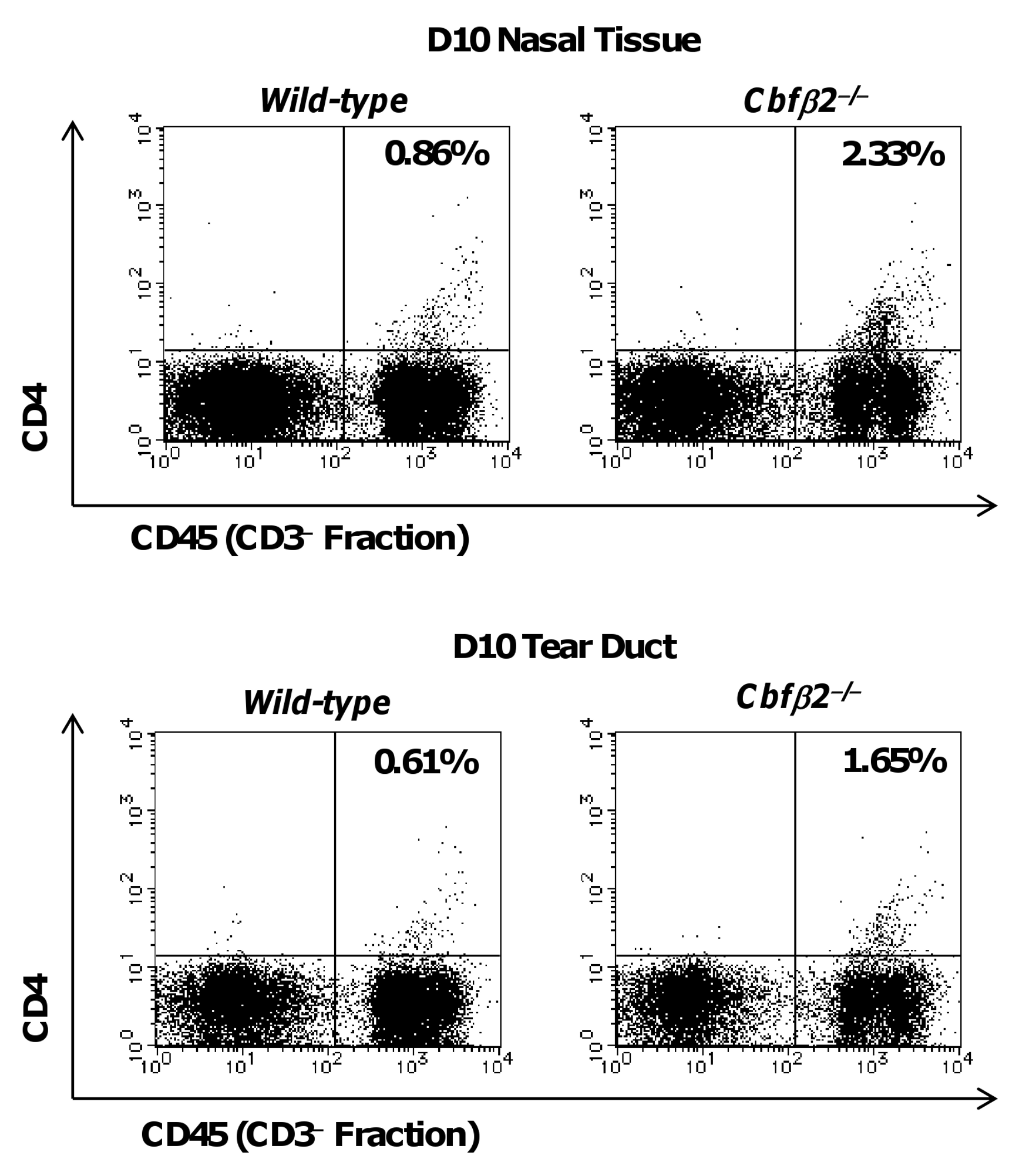

Supplement: S2 Fig — Nasal tissue and tear duct of D10 C57BL/6 wild-type and Cbfβ2 −/− mice were analyzed by means of FACS. CD3−CD4+CD45+ cells were found even in the absence of Cbfβ2. Data shown are Via-prove negative live cell population. Data are representative of at least 3 independent experiments (n = 3 mice/group). (TIF) [file pone.0127460.s002.tif]

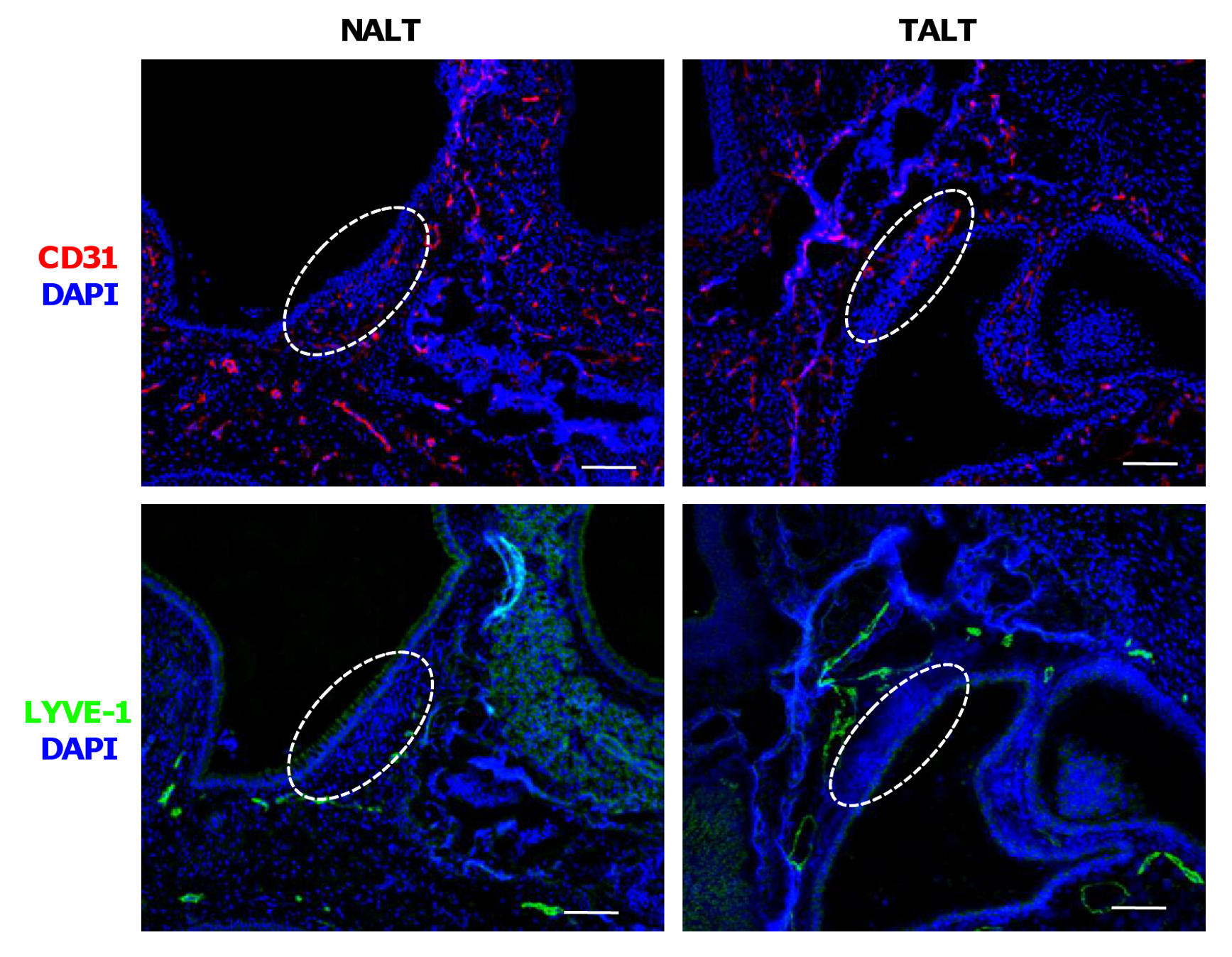

Supplement: S3 Fig — D10 nasal tissue and tear duct of C57BL/6 wild-type mice were examined by means of fluorescence microscopy. CD31 and LYVE-1 positive signals were detected in the area of nasal mucosa and tear duct lamina propria. Dotted lines indicate the site of NALT and TALT anlagens. Data are representative of at least 2 independent experiments (n = 3 mice). (TIF) [file pone.0127460.s003.tif]

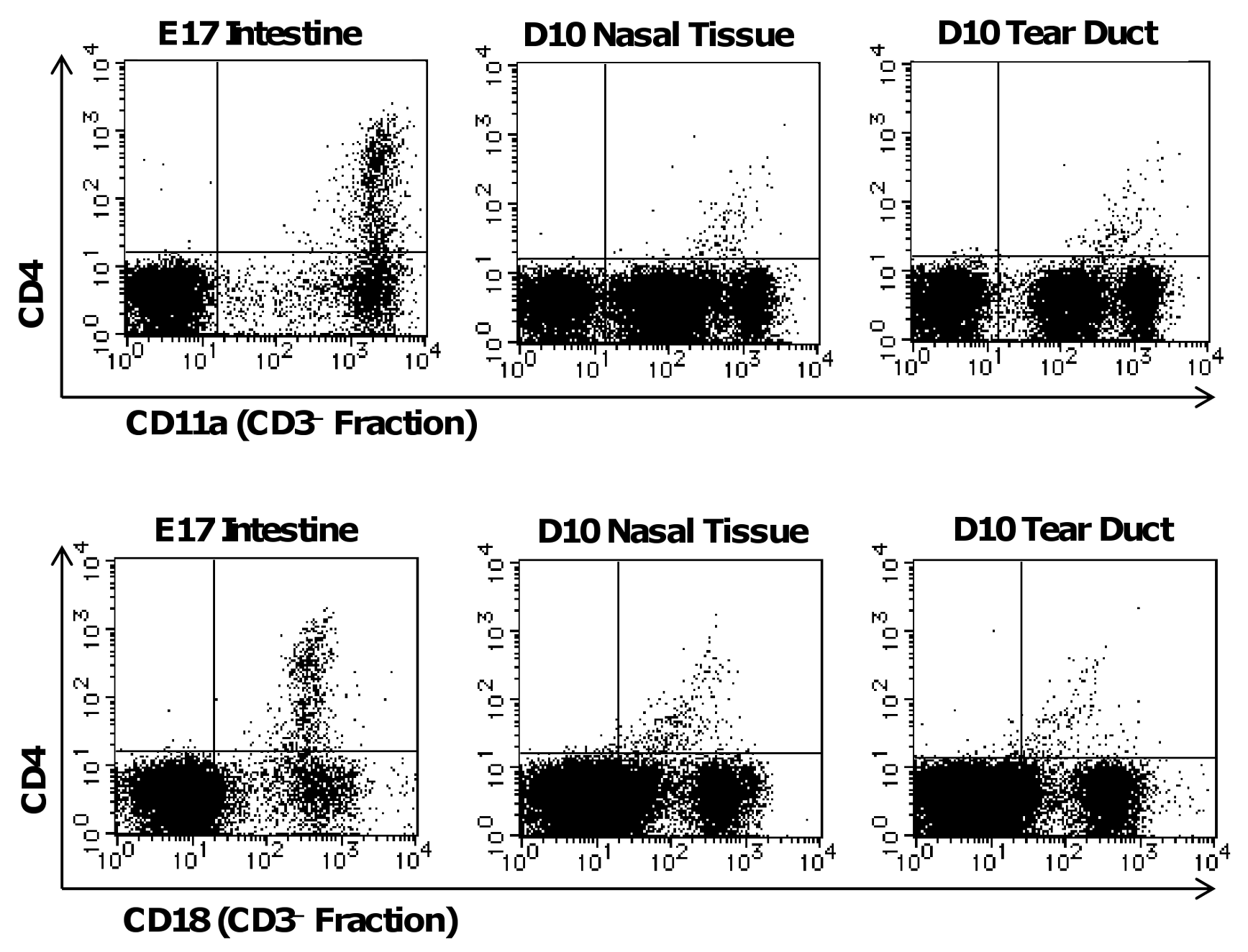

Supplement: S4 Fig — FACS analysis of E17 intestine, D10 nasal tissue, and D10 tear duct was performed to examine the expression of CD11a and CD18 by PPi, NALTi, and TALTi cells, respectively. CD3−CD4+ cells in all three tissue types were found to express CD11a and CD18. Data shown are Via-prove negative live cell population. Data are representative of at least 2 independent experiments (n = 6 mice/group). (TIF) [file pone.0127460.s004.tif]

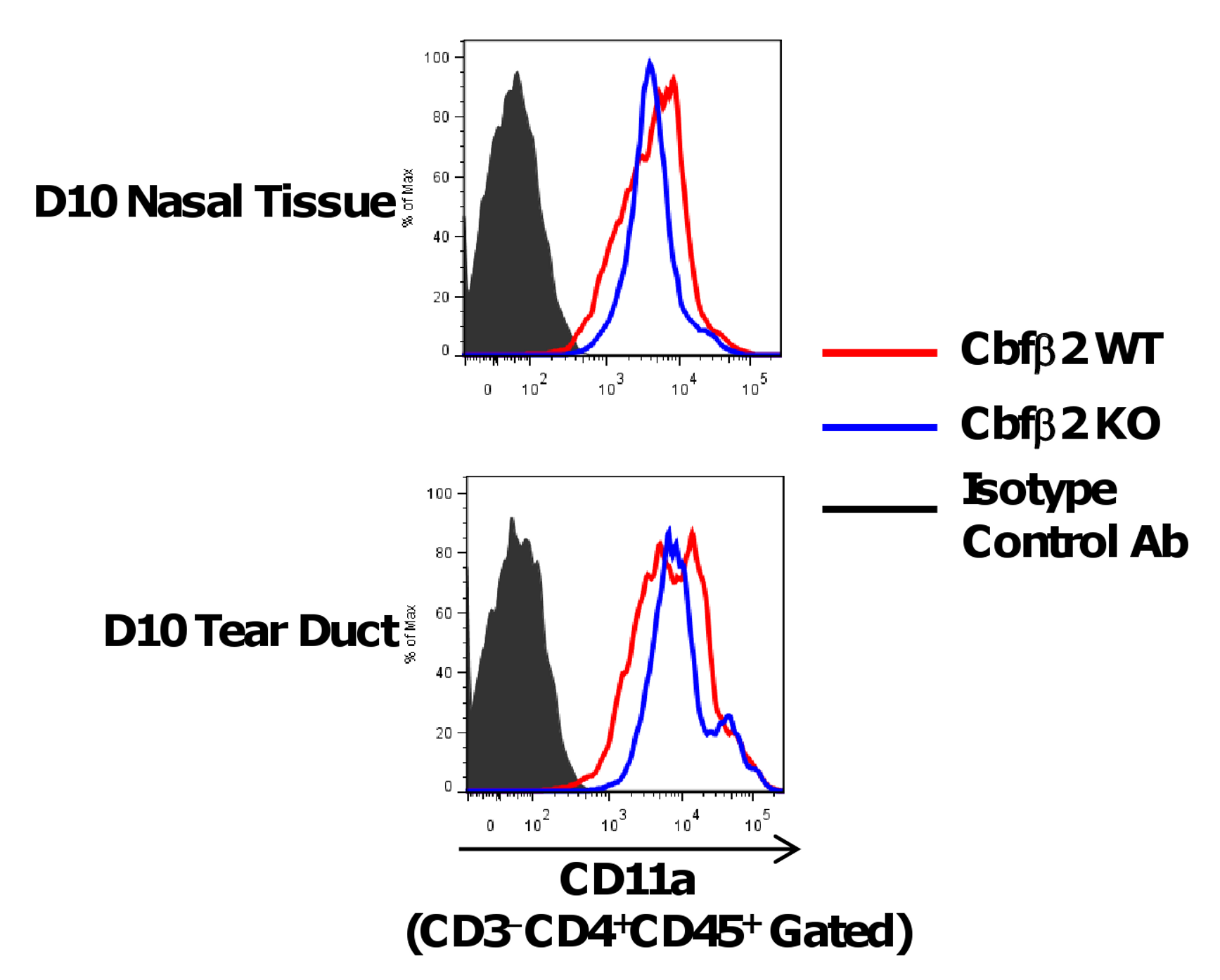

Supplement: S5 Fig — FACS analysis of D10 nasal tissue and D10 tear duct of wild-type mice and Cbfβ2 −/− mice was performed to examine the expression level of CD11a. Histgram data shown are Via-probe−CD3−CD4+CD45+ gated population. The expression level of CD11a in NALTi and TALTi cells of Cbfβ2 −/− mice were not changed as compared with that of wild-type mice. Data are representative of at least two independent experiments (n = 6 mice/group). (TIF) [file pone.0127460.s005.tif]
